# Cross-Neutralization between Bovine Viral Diarrhea Virus (BVDV) Types 1 and 2 after Vaccination with a BVDV-1a Modified-Live-Vaccine

**DOI:** 10.3390/vaccines11071204

**Published:** 2023-07-05

**Authors:** Geromine Grange, Marie Mindeguia, Philippe Gisbert, Gilles Meyer

**Affiliations:** 1 VetHippo’Dome, 63720 Venezat, France; geromine.grg@gmail.com; 2 Clinique Vétérinaire Amikuze, 64120 Béhasque-Lapiste, France; marie.mindeguia@gmail.com; 3 CEVA Santé Animale, 33500 Libourne, France; philippe.gisbert@ceva.com; 4 Interactions Hôtes-Agents Pathogènes (IHAP), Université de Toulouse, INRAE, ENVT, 31100 Toulouse, France

**Keywords:** pestivirus, Bovine Viral Diarrhea Virus, vaccine, neutralizing antibodies, cross neutralization

## Abstract

Control of Bovine Viral Diarrhea Virus types 1 and 2 (BVDV-1 and BVDV-2) involves removing persistently infected animals from the herd, ensuring the biosecurity level of the farms and vaccination for the prevention of fetal infection. Given pestiviruses high genetic and antigenic diversities, one challenge for a BVDV vaccine is to provide the broadest possible heterologous protection against most genotypes and sub-genotypes. The Modified-Live Mucosiffa^®^ vaccine, which contains the BVDV-1 sub-genotype 1a (BVDV-1a) cytopathic Oregon C24 strain, was shown to protect fetuses of pregnant heifers against a challenge with a BVDV-1f Han strain. In this study, we tested the cross-neutralizing antibody (NA) response of 9 heifers at 28, 203- and 363-days post-vaccination with Mucosiffa^®^ against recent and circulating European strains of BVDV-1a, -1b, -1e, -1f and BVDV-2a. We showed that Mucosiffa^®^ vaccination generates a stable over time NA response against all BVDV strains. NA response was greater against BVDV-1a and -1b, with no significant differences between these sub-genotypes. Interestingly the NA response against the two BVDV-2a strains was similar to that observed against the BVDV-1f Han strain, which was the challenge strain used in fetal protection studies to validate the Mucosiffa^®^ vaccine. These results suggest that Mucosiffa^®^ vaccination provides humoral cross-immunity, which may protect against BVDV-1 and BVDV-2a infection.

## 1. Introduction

Bovine Viral Diarrhea Virus (BVDV) is a widespread and economically essential cattle pathogen that belongs to the *Pestivirus* genus of the *Flaviviridae* family. The *Pestivirus* genus comprises eleven recognized species, from Pestivirus A to K [1]. According to this new classification, BVDV belongs to two species, Pestivirus A for the former genotype 1 (BVDV-1) and Pestivirus B for genotype 2 (BVDV-2). The former classifications have been retained in this publication because they are still widely used. Pestiviruses were characterized by a very high genetic diversity which has made it possible to classify BVDV-1 and BVDV-2 into sub-genotypes, at least 24 (BVDV-1a–1x) and 4 (BVDV-2a–2d) for BVDV-1 and BVDV-2, respectively [2,3,4]. Several European countries are currently involved in a BVD control plan, mainly based on the rapid elimination of persistently infected calves (PI), biosecurity measures and sometimes vaccination of susceptible animals. Vaccination is done with either life or killed BVDV vaccines with a fetal protection claim and to protect young cattle against BVDV horizontal infection, especially in the context of the bovine respiratory disease complex. Modified live vaccines (MLV) have been shown to induce both humoral neutralizing antibody and T cell-mediated immune responses, with solid fetal protection for some [5,6,7]. MLV also provides a longer protection duration from clinical disease than inactivated vaccines [8]. Most documented results about BVDV vaccine-induced immunity concerned the humoral response, especially neutralizing antibodies [9,10], which target envelope glycoproteins E1 and E2, with E2 being immunodominant [11,12]. Given pestiviruses’ high genetic and antigenic diversity, especially for glycoprotein E2, one challenge for a BVDV vaccine is to provide the broadest possible heterologous protection against most genotypes and sub-genotypes. Generally, the antibody protection of vaccines is superior against homologous than heterologous genotypes [13,14,15,16]. As traditional French commercial vaccines contain BVDV strains of genotypes 1a, 1b and 2a [17], the question arises regarding their efficacy in protecting against circulating sub-genotypes other than those in them fully. In France during the years 2018 to 2020, the most frequent field samples were of genotypes 1e (57.9%, 146 samples), 1b (32.4%), then 1d (3.4%) and 1l (2.1%) [18]. No BVDV-2 isolate was identified during this period. 

The MLV Mucosiffa^®^ vaccine currently used in Europe contains the BVDV-1 cytopathic Oregon C24 strain of sub-genotype 1a. This vaccine was experimentally shown to fully protect fetuses of pregnant heifers against a challenge with a BVDV-1f Han strain [19,20]. It is also recommended to prevent clinical signs and mortality after transient BVDV infection, especially in the BRD context. Contradictory results have been shown regarding cross-protection between BVDV-1a and BVDV-1b. While some studies showed high levels of cross-reactivity [14,16,21], other studies suggested moderate to insufficient protection [15,16,22,23], depending on the strains used in vaccines and cross-protection studies. A recent study indicated a cross-neutralizing reactivity of Mucosiffa^®^ induced antibodies against a heterologous BVDV-1b Italian strain but, at the same time, a complete absence of cross-neutralization against an Italian BVDV-1e strain [16]. In addition, antibodies induced by Mucosiffa^®^ vaccination were never tested against BVDV-2 strains. Thus, the objective of the present study was to verify whether animals immunized with Mucosiffa^®^ were able to develop a neutralizing antibody (NA) response, 28, 203 and 363 days after vaccination, against viral sub-genotypes of BVDV-1 (including two strains of 1b and 1e sub-genotypes) and BVDV-2 circulating in Europe. 

## 2. Materials and Methods

### 2.1. Virus and Inoculum

Six BVDV strains were tested for neutralization. The BVDV-1a cytopathic (cp) NADL strain belongs to the same sub-genotype 1a as the Mucosiffa^®^ vaccine cp Oregon C24 strain and is also routinely used in numerous serum neutralization (SN) studies. Antibodies generated by the Mucosiffa^®^ vaccine have already been shown to neutralize this strain [16]. The BVDV-1f Han non-cytopathic (ncp) strain was previously used as the challenge strain in the in vivo fetal protection trials with the Mucosiffa^®^ vaccine [22,23]. The BVDV-1e and BVDV-1b sub-genotypes are currently the most detected strains in France [18]. Furthermore, these two sub-genotypes have shown a significant difference in the neutralization efficiency of antibodies generated by the Mucosiffa^®^ vaccine [16]. The BVDV-1b and BVDV-1e ncp stains strains were kindly provided by Dr Ana Moreno, Istituto Zooprofilattico Sperimentale della Lombardia ed Emilia Romagna, Brescia, Italy). The BVDV-2 ncp strains 10-113 (BVDV-2a 10) and 13-035 (BVDV-2a 13) were isolated in France (departments of Nievre and Meuse) in 2010 and 2013, respectively and were kindly provided by the Veterinary analysis laboratory LABOCEA (Quimper, France). They were then genetically typed as BVDV-2a sub-genotype (Prof Mutien Garigliany, Dpt of Pathology, Faculty of Veterinary Medicine, Liège, Belgium).

Each strain was amplified by six passages in bovine Madin Darby Kidney cells (MDBK, ATCC CCL-22) in DMEM medium supplemented with 1% penicillin-streptomycin and 10% horse serum. Previously, MDBK cells were shown to be free of BVDV viruses by RT-qPCR (Bio-T kit^®^ BVDV/BDV Universal, Biosellal, Dardilly, France) and from BVDV antibodies (ID Screen^®^ BVD p80 Antibody Competition, Innovative Diagnostics, Grabels, France). Virus titration was performed by 10-fold dilutions of each strain on MDBK (6 duplicates per virus), and detection of the BVDV virus was done by IPMA (see Section 2.2). Viral titers were calculated according to the Spearman-Karber method. 

### 2.2. Antibody Neutralizing Response

Sera were obtained from a previous experiment demonstrating the fetal protection in pregnant heifers challenged with BVDV-1f Hannover (Han) twelve months after one administration of the live-attenuated Mucosiffa^®^ vaccine [23]. They were collected from 9 heifers on days 28, 203 and 363 (just before the challenge) after vaccination. 

SN assays were performed for the 6 BVDV strains and each selected day in a 96-well cell microplate (4 duplicates per sample) with a constant amount of virus (200 TCID_50_ per well) as already described [24,25]. Control positive and negative sera were also included in each batch of tests. For the five ncp strains, infection was detected by immunoperoxidase assay (IPMA) [25]. For the NADL strain, infected cells were detected based on the cytopathic effect and confirmed by IPMA. Virus NA titers were expressed as the effective dose of 50% (ED_50_) calculated by the Spearman-Kärber method. 

### 2.3. Statistical Analyses

Statistical analyses were performed using GraphPad (La Jolla, CA, USA). Logarithmic transformation was applied to fulfill the variances in homogeneity and normality. Data were expressed as arithmetic mean ± standard deviations (SD). A two-way ANOVA with repeated measures (three-factor split-plot ANOVA) was used to analyze SN titers. When the effects of the “day” and “treatment” factors were significant among interactions, a Bonferroni test between contrasts was used to compare the treatments on each day post-challenge. Levels of significance are indicated in Figure 2 when significant. 

## 3. Results 

**Figure 1:**
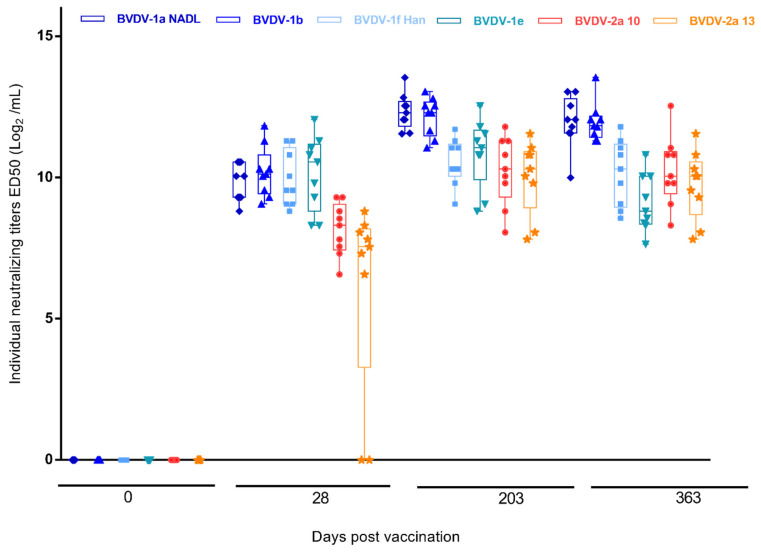
Interleaved box/whiskers (Min. to Max.) showing individual antibody titers at days 28, 203 and 363 post-vaccination (Day 0 just before vaccination) for each virus, expressed as ED50 (Log2)/mL (±sed).

Vaccination with Mucosiffa^®^ induced neutralized antibody response against all tested strains with low intra-group variability. SN stated as early as 28 days after immunization, except for 2 heifers with NA against BVDV-2a 10 but not BVDV-2a 13 strain at this date (Figure 1). However, these two animals had NA to this strain at D203 with similar titers to the other animals. 

**Figure 2:**
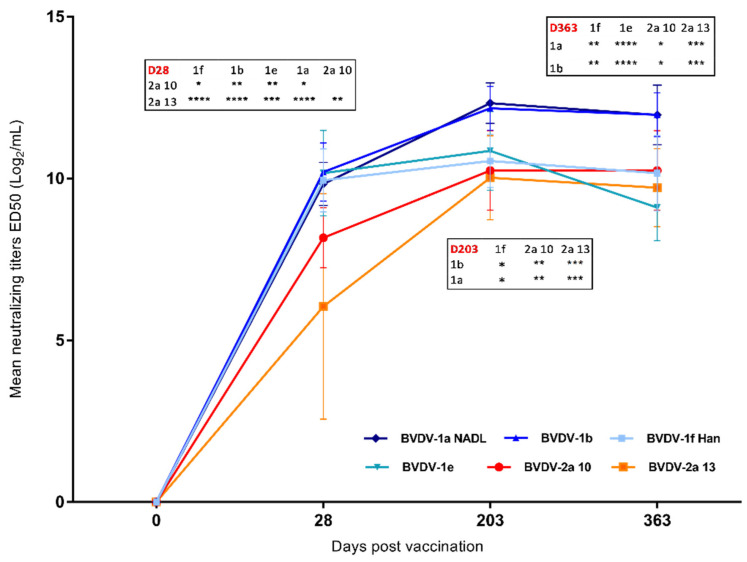
Mean neutralizing antibody titers at days 28, 203 and 363 post-vaccination (Day 0 just before vaccination) for each virus, expressed as ED50 (Log2)/mL (±sed). Tables show significant differences between groups for each day tested: * *p* < 0.05; ** *p* < 0.01; *** *p* < 0.001; **** *p* < 0.0001.

At D28, mean SN titers of all BVDV-1 strains were similar to each other but significantly higher (p between 0.05 and 0.0001) than for genotype 2 strains (Figure 2). In addition, the mean SN titers for BVDV-2a 10 were significantly higher than those neutralizing BVDV-2a 13. After D28, mean SN titers increased for all BVDV strains up to D203 (Figure 2), and then stabilized (BVDV1a, BVDV-1b, BVDV-2a 13, BVDV-2a 10) or dropped slightly (BVDV-1b, BVDV-1e). As we did not sample between D28 and D203, it was impossible to determine exactly when the plateau was reached for each viral strain, most probably before D203. At this date, the mean titers for BVDV-2 strains were similar to those of BVDV-1f Han and BVDV-1e but significantly lower than the mean SN titers of BVDV-1a NADL and BVDV-1b strains (Figure 2). Similarly, the mean titer of BVDV-1f Han was significantly lower than that of BVDV-1a NADL and BVDV-1b, but not BVDV-1e. At D363, titers remained stable, except for BVDV-1e. At this date, the mean titers of NA to BVDV-1b and BVDV-1a NADL were significantly higher than those of all other viruses. In contrast, there were no significant differences in mean NA titers between BVDV-1f Han, BVDV-1e, BVDV-2a 10 and BVDV-2a 13. 

## 4. Discussion 

This study shows that vaccination with Mucosiffa^®^ induces a rapid and stable NA response for at least one year against the various BVDV genotypes tested, despite a slight decrease after D203 for the BVD-1e strain. As in other cross-SN studies [16,24], we observed very low individual variability of NA titers within each group, suggesting homogeneity of BVDV antibody responses in cattle after vaccination with Mucosiffa^®^. The NA response started as early as 28 days after vaccination in almost all heifers. The only exception is the BVDV-2a genotype, where differences were observed at D28 between the two strains, as two cattle in the BDV-2a 13 group had not yet seroconverted. It can be assumed that these two animals rapidly seroconverted after this date as they finally showed similar titers to the other heifers in the group at D203. BVDV-2a 10 and 13 strains were obtained from outbreaks in two French departments, more than 200 km apart, suggesting the circulation of different BVDV-2a viruses in this part of the country in 2010 and 2013. This is confirmed by 5′UTR sequencingand different cross-SN results obtained in this study. However, a recent epidemiological study did not confirm that strains of the BVDV-2a sub-genotype are still circulating in France [18]. In addition, the BVDV-2 prevalence in France is probably low as this study showed the absence of BVDV-2 detection from 211 BVDV-tested isolates circulating in the main cattle breeding French areas between 2018 and 2020 [18]. 

Considering the BVDV-1 genotype, the humoral NA response was greater and more stable against BVDV-1a and -1b, with SN titers increasing significantly from D0 to D203 and remaining steady until the end of the experiment. This seems logical given the phylogenetic proximity between the BVDV-1a NADL and C24 vaccine strain of Mucosiffa^®^. In addition, we found no significant differences between the two BVDV-1a NADL and BVDV-1b groups, suggesting a good cross-neutralization between these two sub-genotypes. This contrasts with other authors who suggested differences in SN titers between BVDV-1a and BVDV-1b, in particular, a low level of antibody response to the BVDV-1b sub-genotype by BVDV-1a vaccines [16,26,27]. The differences can be explained by the high diversity observed in the 1b sub-genotype, probably between the different strains used in these experiments. As the 5′-UTR sequence identity percentage does not necessarily correlate with the antigenic characteristics, this could explain significant antigenic differences observed within the same sub-genotype.

On the other hand, in publications that compared several BVDV vaccines [16,24,27], vaccination with the BVDV-1a Mucosiffa^®^ was shown to induce cross SN against the BVDV-1b strains tested without significant differences. However, titers were higher against homologous BVDV-1a strains. More detailed data on sequence homologies and coefficients of antigenic similarity (R) [28] between the vaccine BVDV-1a C24 strain of Mucosiffa^®^ vaccine and different BVDV-1b strains would provide a better understanding of the cross-protection relationships between these two sub-genotypes. This is all the more important than in Europe. The sub-genotype 1b circulates frequently, while sub-genotype 1a is rarely identified [2,18]. 

Conversely to BVDV-1a and -1b, the titers of BVDV-1f Han and BVDV-1e strains increased until D28 but then remained stable (BVDV-1f) or declined slightly (BVDV-1e) from D203, suggesting a greater antigenic distance with the BVDV-1a vaccine strain. Bachofen et al. [29] already reported R values indicative of significative antigenic difference (R < 25) between sub-genotypes 1e and both 1a and 1b. In addition, Sozzi et al. [16] showed a complete absence of cross-NA response against a BVDV-1e strain in cows immunized with four different vaccines containing BVDV-1a or BVDV-1b strains, including Mucosiffa^®^. This differs from our study, where we find a cross-SN response against BVDV-1e, although less important. This difference could be explained by the SN method used (different) and/or the nature of the virus strain used for infection. In the study of Sozzi et al. [16], the BVDV-1e strain was chosen because the prediction of the antigenic sites carried out in silico indicated a marked antigenic difference compared to the other sub-genotypes. As sub-genotype 1e includes strains that are quite distant phylogenetically [30], the data of Sozzi et al. [16] are not sufficient to generalize this finding to all the BVD-1e strains. In addition, as the SN response was not tested after 28 days of vaccination [16], it is impossible to know whether a NA response might not have occurred later, as was observed for two heifers in the BVDV-2a 13 group of our study. 

Considering cross-neutralization against BVDV-2, we found a lower SN response for BVDV-2a 10 and 13 strains than against BVDV-1a and BVDV-1b, with statistically significant values for the three dates tested. These results are consistent with those of Hamers et al. [24], who showed that the mean titers of NA of 15 BVDV-1 strains were significantly (*p* < 0.05) higher than those neutralizing 7 strains of BVDV-2. This also correlates with a greater genetic distance, especially on the E2 gene supporting humoral immunity [31]. However, an interesting point concerns the evolution of the mean titers of the two BVDV-2a strains and the BVDV-1e and 1f strains. From D203 onwards, we no longer observe significant differences between these 4 viruses. Especially vaccination induced antibodies neutralizing the BVDV-2a strains with the same efficiency as the BVDV-1f Han strain, which was the challenge strain used in fetal protection studies using the Mucosiffa^®^ vaccine [22,23]. In the study for Mucosiffa^®^ official validation for fetal protection [23], all heifers, challenged 363 days after vaccination and corresponding to 87 days of pregnancy, were fully protected against fetal infection and PI production. These results suggest that Mucosiffa^®^ vaccination provides humoral cross-immunity, which may provide fetal protection against both BVDV-1 and BVDV-2a infections. On the other hand, it should be noted that this study did not investigate the innate and adaptive cellular immune response, which, for the MLV vaccine, also contributes to fetal protection against BVDV [30]. 

To date, there are no studies and no scientific consensus to clearly define a threshold of NA at which animals would be protected against BVDV infection. Beer et al. [32] showed that an SN titer of 1:512 was necessary for marked protection against BVDV infection in cattle, although protective effects appear as early as 1:256 [31]. Ridpath et al. [33] set the minimum cross-protection threshold between BVDV-1 and BVDV-2 in 1/16. On this basis, most studies considered antibody titers > 1/20 as indicative of cross-protection. Using an SN method very similar to ours but with different strains, Hamers et al. [24] obtained varied cross-average SN titers (between 2.6 and 8.8 Log2/mL) lower than those obtained in our study. Unfortunately, antibody titers were not associated with a protection criterion in this study. Using our SN test, protection of all cell culture wells at 1:512 [32] would correspond to a titer of 13.7 Log2 ED50/mL, higher than the vast majority of titers obtained in this study, from 6.5 to 13.5 Log2/mL. On the other hand, using an identical SN method to the one used in this work, the full fetal protection against the BVDV-1f Han strain challenge was demonstrated for pregnant cows with SN titers between 8.5 and 9.2 Log2 ED50/mL [22], well below the threshold proposed by Beer et al. [33]. Based on this fetal protection study [22] and setting an arbitrary threshold of 8.5 Log2 ED50/mL for protection, mean SN titers obtained in the present study are all similar or above this threshold for all BVDV-1 and BVDV-2 strains, except at D28 for the BVDV-2a 13 group. 

In conclusion, this study shows that vaccination with Mucosiffa^®^ generates a NA response to BVDV-1 and BVDV-2 heterologous strains. The similarity of the SN responses between BVDV-2a and BVDV-1f Han (challenge strain used in Mucosiffa^®^ validation for fetal protection) strains suggests that Mucosiffa^®^ vaccination provides humoral cross-immunity, which may protect against fetal infection by BVDV genotypes 1 and 2. Finally, a BVDV-2a standardized challenge of pregnant cows previously vaccinated with Mucosiffa^®^ would confirm the cross-fetal protection. 

## Data Availability

Not applicable (data provided in Figure 1 and Figure 2).
